# Responsible Genes for Neuronal Migration in the Chromosome 17p13.3: Beyond *Pafah1b1(Lis1)*, *Crk* and *Ywhae(14-3-3ε)*

**DOI:** 10.3390/brainsci12010056

**Published:** 2021-12-30

**Authors:** Xiaonan Liu, Sarah A. Bennison, Lozen Robinson, Kazuhito Toyo-oka

**Affiliations:** 1 Department of Pharmacology and Physiology, Drexel University College of Medicine, Philadelphia, PA 19129, USA; xl387@drexel.edu; 2 Department of Neurobiology and Anatomy, Drexel University College of Medicine, Philadelphia, PA 19129, USA; sb3776@drexel.edu (S.A.B.); lsr52@drexel.edu (L.R.)

**Keywords:** neuronal migration, chromosome 17p13.3, lissencephaly, Miller–Dieker syndrome, *PAFAH1B1* (LIS1), *YWHAE* (14-3-3ε), *CRK*

## Abstract

The 17p13.3 chromosome region is often deleted or duplicated in humans, resulting in severe neurodevelopmental disorders such as Miller–Dieker syndrome (MDS) and 17p13.3 duplication syndrome. Lissencephaly can also be caused by gene mutations or deletions of a small piece of the 17p13.3 region, including a single gene or a few genes. *PAFAH1B1* gene, coding for LIS1 protein, is a responsible gene for lissencephaly and MDS and regulates neuronal migration by controlling microtubules (MTs) and cargo transport along MTs via dynein. CRK is a downstream regulator of the reelin signaling pathways and regulates neuronal migration. *YWHAE*, coding for 14-3-3ε, is also responsible for MDS and regulates neuronal migration by binding to LIS1-interacting protein, NDEL1. Although these three proteins are known to be responsible for neuronal migration defects in MDS, there are 23 other genes in the MDS critical region on chromosome 17p13.3, and little is known about their functions in neurodevelopment, especially in neuronal migration. This review will summarize the recent progress on the functions of LIS1, CRK, and 14-3-3ε and describe the recent findings of other molecules in the MDS critical regions in neuronal migration.

## 1. Introduction

The formation of the human brain requires the accurate completion of neurogenesis, neuronal migration, and the formation of appropriate connections of more than 100 billion neuronal cells during brain development. Neuronal migration in the developing cortex is composed of multiple steps, including multipolar migration, locomotion, and terminal translocation, and all steps are essential for creating a functional brain. If neuronal migration is disrupted, it results in a wide range of diseases, including brain morphological disorders such as Miller–Dieker syndrome (MDS), epilepsy, and neuropsychiatric disorders such as schizophrenia, attention-deficit hyperactivity disorder (ADHD), and autism spectrum disorder (ASD) [1,2,3,4,5,6,7,8,9]. Lissencephaly is a human brain malformation characterized by a smooth cerebral surface where the characteristic gyral folding pattern of the cerebral cortex is reduced or absent. Isolated lissencephaly sequence (ILS) is a heterogeneous disorder consisting of lissencephaly with no other major malformations. Causative mutations and deletions in *PAFAH1B1* (also known as *LIS1*) as well as centromeric chromosome deletions in 17p13.3 account for most cases of ILS [10]. Mutations in other chromosome regions have also been associated with ILS, such as *RELN* on human chromosome 7q22.1, *DMRTA2* on 1p32.3, and *TUBA1A* (tubulin alpha 1a) on 12q13.2 [11,12,13,14]. X-linked lissencephaly results from mutations in X chromosome-residing genes—the Aristaless-related homeobox gene (*ARX*), and doublecortin (*DCX*)—which are the two most notable X-linked genes causing lissencephaly [15]. By contrast, MDS consists of severe classical lissencephaly associated with other symptoms, such as facial anomalies, and MDS patients have larger heterozygous deletions that include the *PAFAH1B1* (*LIS1*) and *YWHAE (14-3-3*ε*)* genes, compared with patients with ILS. The 17p13.3 region is well conserved, and genes within the human 17p13.3 chromosome region are syntenic to mouse chromosome 11 [16,17]. A previous study clarified that an approximate 1.4-M deletion region (MDS critical region) from the *PAFAH1B1* gene to the *YWHAE* gene in the 17p13.3 region is critical for the development of MDS [18].

Mechanistically, regulation of MTs and F-actin organization, as well as regulation in cell adhesion and neurotrophic stimulation, is essential for neuronal migration, and the coordination between these mechanisms makes it possible for the neurons to migrate. Cortical neurons typically migrate by two modes of migration: radial migration and tangential migration. Some neurons also display more complicated migration called switching migration [19]. In lissencephaly, radial migration defects account for the majority of the cause, although defects in tangential migration are also implicated [15]. During migration, neurons polarize to form a leading process and a trailing process. The tip of the leading process is enriched in F-actin and actin motor proteins (i.e., myosins), whereas the stem region of the leading process is enriched with MTs. In locomotion, post-mitotic neuron migration occurs along the radial glial cells, and adhesion molecules help neurons to attach to the radial glial cells, creating traction force for the movement. MTs form a cage-like structure surrounding the nucleus, which helps the nucleus move during migration [20]. The retraction of the trailing process in migrating neurons is mainly mediated by actomyosin but is also mediated by MTs [21].

**Figure 1:**
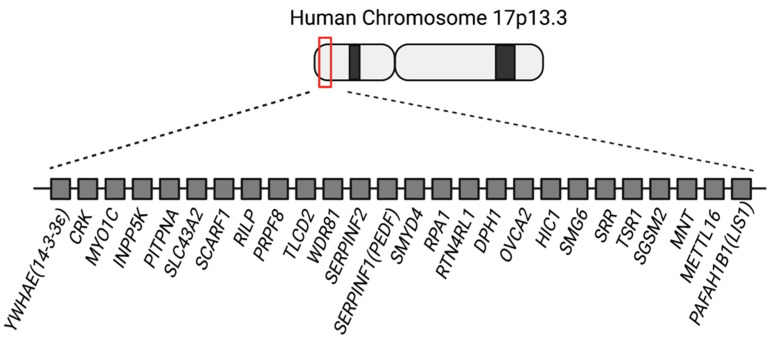
Schematic illustration of the 26 genes in the human chromosome 17p13.3 Miller–Dieker syndrome critical region.

There are 26 genes from *PAFAH1B1* to *YWHAE* in the human chromosome 17p13.3 region that are deleted in MDS patients (Figure 1). So far, 3 out of the 26 genes (*PAFAH1B1*, *CRK*, and *YWHAE*) in the MDS critical region have been widely known to be involved in cortical development, including neuronal migration [18,22,23]. *Pafah1b1*, *Ywhae*, and *Crk* knockout mice display cortical dysplasia. In patients, multiple genes in the 17p13.3 region are disrupted. Therefore, it is difficult to prove which genes are involved in neuronal migration defects in addition to these three genes. Patients with either ILS or MDS have a heterozygous loss of the *PAFAH1B1* gene in the 17p13.3 locus and display combinatorial defects, including neuronal migration defects, epilepsy, and craniofacial defects. However, brain malformation is the most characteristic and prominent phenotype in ILS and MDS patients. *PAFAH1B1*, *YWHAE*, and *CRK* genes are involved in brain malformation caused by the neuronal migration defects observed in MDS patients. However, it is unclear if the other 23 genes are also important for proper cortical formation and involved in brain malformation in MDS patients. Therefore, it is important to fill gaps in our knowledge about the functions of the remaining 23 genes in cortical development. The purpose of this review paper is to summarize the knowns of the three most notable genes—*PAFAH1B1*, *YWHAE*, and *CRK*—and to collect the pieces related to neuronal migration in the other genes in the 17p13.3 region since these have not gained much attention previously. This will shed light upon the etiology of MDS as well as the fundamental mechanisms of cortical development.

## 2. The Roles of *YWHAE*, *PAFAH1B1*, and *CRK* in Neuronal Migration

### 2.1. YWHAE/14-3-3ε

**Figure 2:**
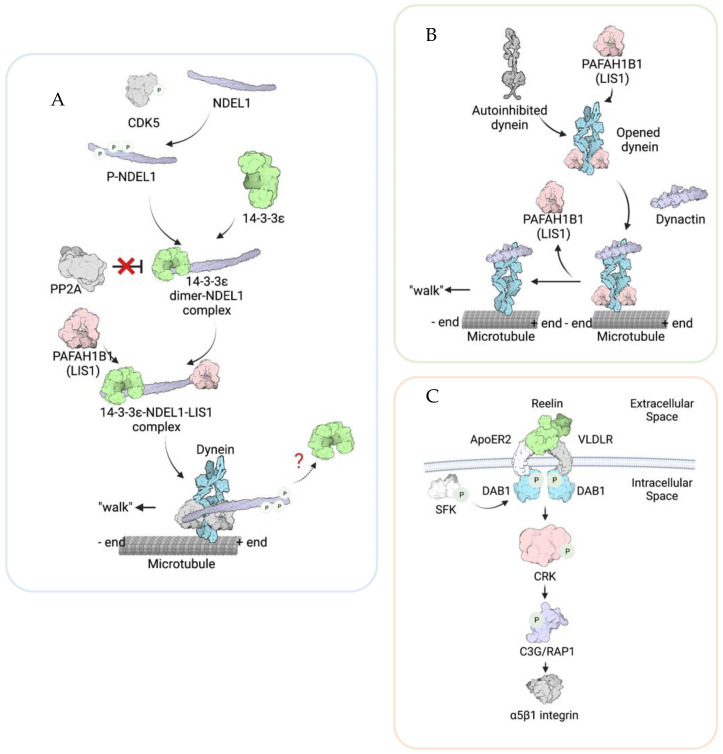
Schematic diagrams illustrating the molecular mechanisms of PAFAH1B1 (LIS1), CRK, and 14-3-3ε in regulating neuronal migration. (**A**) Phosphorylated NDEL1 (NUDEL) protected by 14-3-3ε from dephosphorylation. NDEL1 is phosphorylated by CDK5. Once phosphorylated, 14-3-3ε interacts with NDEL1 and protects it from the protein phosphatase 2 (PP2A)-mediated dephosphorylation. Therefore, NDEL1 could stay activated. Active NDEL1 transiently interacts with PAFAH1B1 (LIS1) and helps PAFAH1B1 (LIS1) bind to dynein, therefore promoting the movement of dynein along microtubules (MTs). However, it is unclear whether 14-3-3ε stays with dynein in the MTs. (**B**). PAFAH1B1 (LIS1) promotes dynein “open” conformation. When dynein is in an autoinhibited state, it has a low microtubule on rate and a reduced ability to bind to dynactin. PAFAH1B1 (LIS1) binds to “open” state dynein and stabilizes dynein at the “open” state. This allows dynein to stay at a high microtubule-binding status. Afterward, PAFAH1B1 (LIS1) dissociates from the dynein–dynactin complex. In this way, PAFAH1B1 (LIS1) promotes microtubule mediate migration by regulating dynein function. (**C**) CRK plays an essential role in the Reelin signaling cascade. Reelin pathways are activated by Reelin interacting with the transmembrane Reelin receptors, ApoRE2, and VLDLR. This triggers the phosphorylation of Dab1 by SFK at the inner leaflet of the plasma membrane. DAB1 then recruits CRK and activates CRK by phosphorylation. Activated CRK promotes the activation of the Crk SH3-binding guanine nucleotide-releasing/exchange factor (C3G)/Ras-proximate-1 (RAP1) pathway, which activates the cell matrix adhesion molecule α5β1 integrin, thus promoting cell migration. The illustrations were created with BioRenders.com (7 December 2021).

14-3-3 proteins are conserved and ubiquitously-expressed [24]. The 14-3-3 family is composed of seven isoforms in mammalian cells (denoted by Greek letters β, γ, ε, η, σ, τ, and ζ). These 14-3-3 proteins bind to more than 100 targets and are involved in multiple cellular functions, including cell cycle control, apoptosis, and cancer [24]. The protein 14-3-3ε is coded by a gene, *YWHAE* (Tyrosine 3-Monooxygenase/Tryptophan 5-Monooxygenase Activation Protein Epsilon), located in the 17p13.3 chromosome region. Mechanistically, 14-3-3ε regulates MT organization via protection of the nuclear distribution protein nude-like 1 (NDEL1, formerly known as NUDEL) from postmodification (Figure 2A). The 14-3-3ε protein interacts with the NDEL1, and this binding prevents phospho-NDEL1 from dephosphorylating by protein phosphatase 2 (PP2A). Thus, NDEL1 stays active and positively regulates the interaction between LIS1 and dynein. We have previously demonstrated that *14-3-3*ε is a critical factor in neuronal migration, indicating that the *YWHAE* gene is a strong candidate as one of the major genes responsible for the more severe lissencephaly phenotype displayed by MDS patients [25]. Using human patient samples, we also demonstrated a strong correlation between deficiency of the *YWHAE* gene and the severity of cerebral cortical malformations in MDS patients [18]. In addition to the 14-3-3ε functions in locomotion, 14-3-3ε regulates earlier cortical development, including neurogenesis, neuronal differentiation, and the distribution of intermediate progenitor cells (IPCs), suggesting the importance of 14-3-3ε in multipolar migration [26], and 14-3-3ε KO resulted in an increased number of proliferating progenitor cells and a broader distribution of the cells in the cortex, as well as an altered progenitor cell differentiation into neurons in the mouse model.

*YWHAE* is also implicated in the 17p13.3 microduplication syndrome [27]. It has been reported that microduplications in the 17p13.3 region result in a new genetic syndrome termed “the 17p13.3 microduplication syndrome”, which is associated with neurological disorders [28,29,30,31,32,33,34,35,36]. Specifically, 17p13.3 microduplication syndrome is characterized by various-sized duplications in the 17p13.3 chromosome locus, resulting in severe developmental defects, including autism spectrum disorder (ASD), epilepsy, intellectual disabilities, and malformation in the hands and feet. Importantly, the microduplication minimal region, a 72kb region within the 17p13.3 locus, has been defined, and this region exclusively contains the *YWHAE* gene encoding the 14-3-3ε protein and is strongly associated with ASD [30,33]. Although no pathological analyses have been performed in patients with 17p13.3 microduplication syndrome before, ASD patients show defects in neuronal morphogenesis, including neurite formation and spine formation [37,38,39]. Therefore, the defects in neuronal morphogenesis and spine formation and synaptogenesis caused by *YWHAE* overexpression may be an associated cause of ASD seen in patients with this syndrome and provide the first pathological findings. These studies strongly implicate *YWHAE* as a causative gene for ASD. We confirmed that *Ywhae* overexpression resulted in defects in neurite initiation during cortical development in vivo, mechanistically involving the aforementioned X-linked lissencephaly-associated gene *DCX* [40]. In addition, an association of *YWHAE* with schizophrenia has been recently recognized [41]. Interestingly, the 14-3-3 protein family, which consists of 7 isoforms, has been implicated in schizophrenia, ADHD, and general brain development, either when disrupted individually or together. This poses a possibility of cross-talk between the 14-3-3 isoforms. For instance, expression alteration in 5 out of 7 14-3-3 family members—*YWHAB* (14-3-3β), *YWHAE*, *YWHAH* (14-3-3η), *YWHAZ* (14-3-3ζ), and *SFN* (14-3-3σ)—is seen in cases of ASD and schizophrenia [41]. Moreover, the *Ywhae/Ywhaz* double KO mice show more defects in neuronal migration and additional disruptions in other neurodevelopmental stages, such as proliferation in neuronal progenitor cells compared with single KO *Ywhae* or *Ywhaz* in mice, suggesting a strong genetic interaction between *YWHAE* and *YWHAZ* [25,26,42].

Hence, 14-3-3ε is a gene responsible for MDS by regulating neuronal migration but is also implicated in multiple steps of cortical development, including neurogenesis, neuronal differentiation, and neuronal morphogenesis.

### 2.2. PAFAH1B1 (LIS1)

The *PAFAH1B1* (*LIS1*) gene localizes at the telomeric region of chromosome 17p13.3 [30,43]. The implication of *PAFAH1B1* in neuronal migration and Lissencephaly/MDS has been extensively analyzed and reported [44,45,46,47,48,49,50]. PAFAH1B1 was first identified as a subunit of platelet-activating factor acetylhydrolase (PAF-AH) [51], and functions as a regulator of microtubule (MT) motor proteins have been reported [52]. Point mutations and intragenic deletions in *PAFAH1B1* were identified in ILS patients who showed no gross structural chromosomal rearrangements [53,54]. Although the more severe neuronal migration phenotype displayed by MDS patients suggests that genes other than *PAFAH1B1* are responsible for this phenotype, *PAFAH1B1* is a major responsible gene for lissencephaly phenotypes seen in MDS patients. In addition, *PAFAH1B1* mutations in humans and mice result in seizures, and *PAFAH1B1*-deficient mice show sociability defects related to ASD but not the repetitive behavior, implicating *PAFAH1B1* in the proper establishment of neural circuits in addition to neuronal migration [55,56,57,58,59].

Studies about PAFAH1B1 functions in dynein regulation not only in neurons but also in other cell types have been extensively performed, and numerous pieces of evidence have been accumulated [47,50,60]. Previous studies suggest multiple functions of LIS1 in dynein regulation (Figure 2B). For example, PAFAH1B1 is important for binding to MTs and dissociating dynein from MTs [61,62]. The structural analysis of PAFAH1B1 functions in dynein also revealed that PAFAH1B1 induces a persistent microtubule-bound state in dynein by acting on its linker domain [63]. Furthermore, PAFAH1B1 proteins seem to be dissociated from dynein after the dynein–dynactin complexes are composed [64]. In addition to the PAFAH1B1 functions in MTs and dynein, recent studies have reported PAFAH1B1 functions in actin regulation [65,66]. In migrating NIH3T3 cells, *Pafah1b1* knockdown resulted in a reduction in traction force and disorganization of the microtubules and actin filaments [67]. This suggests that PAFAH1B1 might regulate actin in addition to MTs, and PAF1H1B1-driven traction force might be essential for neuronal migration.

### 2.3. CRK

The *CRK* gene is also localized in the 17p13.3 region, encodes an adapter protein that binds to tyrosine-phosphorylated proteins, and is involved in the Reelin signaling pathway [68]. Reelin is a secreted extracellular glycoprotein encoded by one of the ILS-associated genes, *RELN*, as we briefly mentioned in the introduction [11]. The reeler mice were first reported in 1951 [69], and their brain defects were extensively characterized [70,71]. Reelin binds to its two receptors: very-low-density lipoprotein receptor (VLDLR) and apolipoprotein E receptor 2 (apoER2). The two receptors then activate the adaptor protein Disabled-1 (Dab1), recruiting and activating the Src family tyrosine kinases (SFK), such as Src and Fyn, as well as interacting with various adaptor proteins, including Crk and Crk-like (CrkL) [72,73,74,75] (Figure 2C). Since homozygous *Crk* and *CrkL* knockout mice are lethal in early development, the functions of Crk and CrkL in neuronal migration were not clear [76,77]. By producing and analyzing the double *Crk* and *CrkL* conditional deficient mice, it has been found that *Crk* and *CrkL* are essential for neuronal migration [77]. After Reelin binds to its receptor, α5β1 integrin is activated through the intracellular pathway involving Dab1, Crk/CrkL, C3G, and Rap1, and this signaling pathway is vital for terminal translocation, the final step of neuronal migration [78,79]. Patients with chromosome 17p13.3 deletions involving *CRK* and *YWHAE* but not *PAFAH1B1* were reported, and they showed generalized epilepsy, growth retardation, and macrocephaly, implicating *Crk* deficiency in epilepsy [80]. Another patient who had 730Kb deletion containing 11 genes in chromosome 17p13.3, including *YWHAE* and *CRK* but not *PAFAH1B1,* showed infantile spasms syndrome [81]. These data suggest that the *CRK* gene is one of the causative genes for more severe neuronal migration defects and may be partially responsible for the seizure phenotypes in MDS patients.

Moreover, in ASD patients, Reelin protein expression is reduced, predicting a downregulation of CRK signaling in ASD [82,83]. Interestingly, *RELN* mRNA is a target of the fragile X mental retardation protein (FMRP), coded by ASD risk gene *FMR1* [84]. Therefore, the reduced RELN may be due to alteration of FMRP expression. *FMR1* mutations result in fragile X syndrome, one of the most common heritable forms of ASD [85]. Neuronal migration and neurite formation defects, as well as altered synaptic plasticity, have been demonstrated in FMRP deficiency models [38,86,87,88,89]. The *Fmr1*-deficient mice display repetitive behavior and sociability defects [90]. Social behavior phenotypes are also observed in different loss-of-function models for Reelin functional study. However, no repetitive behavioral abnormality has been described so far [91,92].

In conclusion, CRK might be also one of the causes of the autistic phenotype in MDS patients and might be functionally associated with FMRP.

## 3. The Functions of Other Genes in the 17p13.3 Region and Their Potential Roles in Neuronal Migration

There are 23 genes in the critical region of chromosome 17p13.3 MDS, aside from the most notable ones (i.e., *PAFAH1B1*, *CRK*, and *YWHAE*). Some of them are involved in non-neuronal cell migration and microtubule and actin regulation (Table 1). Therefore, it could be worthwhile to study them in neuronal migration and dissect their functions in the onset and development of lissencephaly. Here, we focus on four genes that are involved in neuronal development, cytoskeletal organization, or engaging in protein interactions that have been implicated in cell migration.

*SERPHINF1* is a member of the superfamily of serine protease inhibitors (Serpin) encoding the 50-kDa secreted protein pigment epithelium-derived factor (PEDF) [93], which was originally purified from the culture media of human retinal pigment epithelial cells [94]. PEDF is a non-inhibitory serpin, and PEDF acts as a multifunctional factor, such as neurotrophic, anti-tumorigenic, and anti-angiogenic factor [95,96,97]. *Serpinf1* KO mice show multiple defects in the retina, particularly vessel formation [98]. Although the *Serpinf1* gene is localized in the 17p13.3 chromosome region and often deleted or duplicated, resulting in neurodevelopmental disorders, few studies have taken into account the roles of *Serpinf1* (PEDF) in brain development [43]. Our group recently analyzed Pedf’s function in corticogenesis and found that Pedf deficiency by specific shRNA resulted in multiple defects in neuronal development [43]. These include radial glial cell morphogenesis, neuronal migration, neurite formation, and spine formation [43]. We performed in utero electroporation (IUE) at the E15.5 embryos with Pedf-specific shRNA to target the upper layer neurons. Pedf-deficient post-mitotic neurons could migrate and reach the cortical plate (CP), suggesting no effects on multipolar migration from the ventricular zone to the subventricular zone and locomotion from the intermediate zone to the CP. However, Pedf -deficient neurons are sparsely distributed within the CP, suggesting defects in terminal translocation. PEDF is a secreted protein, and four receptors have been identified. They are PEDF-R, ribosomal protein SA (RPSA), plexin domain containing 1 (PLXDC1), and plexin domain containing 2 (PLXDC2) [99,100,101]. PEDF-R, also known as ATGL, is responsible for mediating PEDF’s effect on vascular hyperpermeability and triglyceride degradation, as well as protection against apoptosis induced by glucocorticoid [102,103]. RPSA, previously termed the 37-kDa laminin receptor precursor/67-kDa laminin receptor, is a laminin receptor that contributes to cell adhesion, migration, neurite outgrowth, and a variety of other events. PLXDC1, also known as tumor endothelial marker 7 (TEM7), and PLXDC2, also known as a mitogen for neuroprogenitors, are homologous membrane proteins [104,105]. Rpsa knockdown (KD) by shRNA in combination with IUE at E15.5 resulted in similar results to Pedf KD in neuronal migration and neurite morphogenesis [43]. Rpsa-deficient neurons in the upper cortical layers could migrate the CP but were sparsely distributed in the CP, suggesting defects in the terminal translocation. The lower layer neurons were also analyzed by performing IUE at E13.5 and showed more severe defects in terminal translocation. Thus, the Pedf-Rpsa signaling pathway is important for the proper completion of neuronal migration by regulating terminal translocation during cortical development. This is a new observation that implicates another gene in neuronal migration in addition to three genes: *PAFAH1B1*, *CRK*, and *YWHAE*.

Myosin 1c (MYO1C) is an unconventional myosin belonging to the class one myosin family. MYO1C is composed of three major domains: the N-terminal actin motor domain, regulatory neck domain that can bound to regulatory molecules, and the C-terminal tail domain that contains a pleckstrin homology domain. MYO1C has been indicated to regulate actin organization and lipid transportation, which are two aspects that are important for cell migration. As for actin regulation, MYO1C has been shown to be important for the regulation of cell cortical actin networks. The motor and tail regions of MYO1C cooperate to tether F-actin to the plasma membrane, thereby facilitating the establishment and dynamics of the cell shape. MYO1C not only works as a hook but also as a support point to power the F-actin to slide. Moreover, MYO1C promotes the formation of filamentous actin by transporting the globular actin [106]. On the other hand, the transportation of lipids to the cell membrane provides material for membrane extension. It has been found that MYO1C is involved in lipid recycling by promoting lipids to recycle to the cell membrane in HeLa cells [107]. In addition, the MYO1C PH domain can bind to phosphatidylinositol (4,5)-bisphosphate, which is intensively involved in molecular signals that associate with cell proliferation, polarity, and migration. Although, to the best of our knowledge, no previous studies showed MYO1C being involved in cortical neuronal migration, a selection of papers reported MYO1C regulating migration in tumor cells, such as glioblastoma cells (1321 N1 cell) and endometrial carcinoma cells [108,109].

Scavenger receptor class F member 1 (*SCARF1*), also known as scavenger receptor expressed by endothelial cells (*SREC*), encodes a transmembrane protein of the scavenger receptor family. Although its name implies its expression in endothelial cells, it is present in various cell types, including neuronal cells and epithelial cells [110]. In addition, SCARF1 is widely expressed throughout different tissues, including the brain [111]. The fundamental function of SCARF1 is to mediate the uptake of chemically modified low-density lipoproteins (LDLs) into cells via the extracellular domain. Other functions that have been revealed, although very limited, indicate SCARF1 might be important in neuronal migration. Shibata et al. have reported that the cytoplasmic domain of SCARF1 is important for actin organization via interacting with the actin regulatory protein Advillin [111]. Patten et al. found that SCARF1 acts as a critical adhesion regulator in the endothelial cells and leukocyte adhesive interaction. In this way, SCARF1 regulates leukocyte migration. However, the SCARF1 ligand in leukocytes that mediates this adhesive cascade is still unknown [110]. To the best of our knowledge, SCARF1 has not yet been studied in neuronal migration. However, *SCARF1* could be a strong candidate gene for regulating neuronal migration due to its known functions in actin organization, cell adhesion, and non-neuronal cell migration.

Serine racemase (SRR) is an enzyme that was first discovered in the rodent brain, and it is enriched in glial cultures of the rodent cerebral cortex [112]. SRR is responsible for synthesizing D-serine from L-serine. The former is an agonist for the N-methyl-D-aspartate (NMDA) receptor and is essential in the NMDA signaling. SRR also helps to produce pyruvate and ammonia in the process of D-serine or L-serine alpha through beta elimination, which is a way to dehydrate serine [113]. While generating D-serine, SRR promotes N-methyl-D-aspartate (NMDA) receptor signaling, one of the most important machinery-regulating neuronal activities and neurodevelopmental events. NMDA receptors mediate pathways essential for neuronal migration by activating NMDAR-mediated calcium signaling and activating extracellular signal-regulated kinase (ERK) signaling cascades [114,115]. Aside from the catalytic functions, SRR is also involved in direct interactions with other proteins. One in particular is Disrupted-in-Schizophrenia-1 (DISC1), whose name alludes to its correlation with the neurodevelopmental disease schizophrenia [116]. Indeed, DISC1 was found to be involved in multiple neurodevelopmental events, including neuronal migration [117]. DISC1 plays a determinative role in the radial migration of the hippocampal pyramidal neurons as well as tangential migration of the cerebral cortical interneurons [118,119]. Furthermore, a DISC1 interacting protein, CAMDI, has been shown to regulate radial migration in the cortical neurons [120]. Although the interaction between DISC1 and SRR is important for D-serine synthesis, SRR can also generate agglomerates with DISC1 in cortical neurons, which also promotes NMDAR activity [121]. According to these findings, it is possible that SRR might also be implicated in neuronal migration. However, direct analysis of SRR in neuronal migration has not been performed, leaving a niche in the neuronal migration research field.

## 4. Conclusions

Abnormal neuronal migration is associated with neurodevelopmental diseases, most notably lissencephaly and more severely MDS. In this review, we have discussed the most well-studied three MDS critical genes—*PAFAH1B1*, *CRK*, and *YWHAE*—in the human chromosome 17p13.3, as well as those that have been unexplored in the brain development area, such as *PEDF*, *SCARF1*, *SRR*, and *MYO1C*. It is interesting to find that a lot of the genes have been studied in the migration of many cell types other than neurons. Additionally, some of these genes have been demonstrated in the regulation of cytoskeletal components, namely microtubules and F-actin. Therefore, they pose some level of potential to be involved in neuronal migration. We think this is extremely important, because MDS is not caused by a single gene but rather by a combination of *PAFAH1B1*, *CRK*, and *YWHAE* deletion. In fact, a large portion of the patients have a piece of gene deletion or mutation spanning from *PAFAH1B1* to *YWHAE*, including the under-examined genes. We hope this review can curtain up for studying the other 23 genes in neurodevelopment, especially in the neuronal migration research field.

Current animal models for lissencephaly and MDS are *Pafah1b1*, *Ywhae*, and *Crk* single-KO models and the *Pafah1b1*/*Ywhae* double-KO model [25,49,77]. Models for other genes have been generated, such as *Srr* KO mice, *Serpinf1* KO mice, and *Scarf1* KO mice. Neuronal migration analysis using those single-gene KO mice may help us understand the functions of the other 23 genes in neuronal migration. Human-induced pluripotent stem cells (hiPSCs) derived from MDS patients are currently available, and cerebral organoids derived from the hiPSCs have become a useful model for neuronal migration research [122,123]. Comparisons between hiPSCs derived from patients carrying different MDS critical gene deletions or mutations could become useful for dissecting the functions of each MDS critical gene.

**Table 1:** Genes’ functions and involvements in cell migration.

Gene	Functions of the Protein	Involvement	References
*MNT*	Regulator of the MYC/MAX/MAD network	Affects migration in the human hepatocellular carcinoma (HCC) cells *Mnt* KO mice have craniofacial defects	[124] Wu et al., 2012 [125] Toyo-oka et al., 2004
*SGSM2*	GTPase-activating protein involving in the modulation of the GTPases RAP and RAB	Interacts with E-cadherin and enhances migratory cell adhesion in the human epithelial T47D cells	[126] Lin et al., 2019
*SRR*	Production of D-serine from L-serine	Interacts with Disrupted-in-Schizophrenia-1 (DISC1), and DISC1 KD causes a defect in cortical neuron radial migration	[121] Jacobi et al., 2019
*HIC1*	Transcription repressor and tumor suppressor	Important for cranial neural crest migration via regulating cadherin protein expression pattern and canonical Wnt signaling *Hic1* KO mice show craniofacial defects	[127] Ray et al., 2020 [128] Valenta et al., 2006 [129] Carter et al., 2000
*DPH1 (OVCA1)*	Responsible for diphthamide biosynthesis	*Dph1* KO causes craniofacial abnormalities in mice, but no observations indicate a defect in neuronal migration	[130] Yu et al., 2014
*RTN4RL1 (NGR3)*	Cell surface receptor Regulate the phosphorylation of SRC and FAK	Regulates epithelial cell migration	[131] He et al., 2018
*RPA1*	Replication protein A	Overexpression causes 17p13.3 instability	[36] Outwin et al., 2011
*RILP*	Endocytosis regulator Induces the recruitment of dynein–dynactin to Rab7-containing late endosomes and lysosomes Promote the transport of endosomes and lysosomes along MTs Interact with Ral guanine nucleotide dissociation stimulator	RILP inhibits cell migration in cancer cells	[132] Margiotta et al., 2017 [133] Wang et al., 2015
*SCARF1*	A member of the Scavenger receptor. Regulates endocytosis	Regulates cell adhesion in human endothelial cells. Expresses in the embryonic brain. Interacts with actin-regulatory protein, Advillin in mouse neuroblastoma cell (N2a)	[110] Patten et al., 2017 [111] Shibata et al. 2004
*SLC43A2*	Amino acid transporter for methionine uptake	Essential for mouse embryonic development	[134] Guetg et al., 2015
*MYO1C*	Unconventional actin motor	Regulates cell cortex tension Regulates F-actin polymerization by transporting G-actin at the leading edge of migrating endothelial cells Promotes migration of 1321 N1 glioblastoma cell	[106] Fan et al., 2012 [108] Edimo et al., 2016

## Data Availability

Not applicable.
