# Correction: Liu et al. Responsible Genes for Neuronal Migration in the Chromosome 17p13.3: Beyond *Pafah1b1(Lis1)*, *Crk* and *Ywhae(14-3-3ε)*. *Brain Sci.* 2022, *12*, 56

**DOI:** 10.3390/brainsci12030311

**Published:** 2022-02-25

**Authors:** Xiaonan Liu, Sarah A. Bennison, Lozen Robinson, Kazuhito Toyo-oka

**Affiliations:** 1 Department of Pharmacology and Physiology, Drexel University College of Medicine, Philadelphia, PA 19129, USA; xl387@drexel.edu; 2 Department of Neurobiology and Anatomy, Drexel University College of Medicine, Philadelphia, PA 19129, USA; sb3776@drexel.edu (S.A.B.); lsr52@drexel.edu (L.R.)

The authors wish to correct the following error in this paper [1]. In Figure 2, the full name of C3G is incorrectly stated in the figure legend. The accurate description is: Crk SH3-binding guanine nucleotide-releasing/exchange factor (C3G).

The authors apologize for any inconvenience caused to the readers as a result of this change. The original article has been updated.
